# Bifocal Ultrasound Focusing Using Bi-Fresnel Zone Plate Lenses

**DOI:** 10.3390/s20030705

**Published:** 2020-01-28

**Authors:** Sergio Pérez-López, José Miguel Fuster, Pilar Candelas, Daniel Tarrazó-Serrano, Sergio Castiñeira-Ibáñez, Constanza Rubio

**Affiliations:** Centro de Tecnologías Físicas, Universitat Politècnica de València, 46022 València, Spain; serpelo1@teleco.upv.es (S.P.-L.); pcandelas@fis.upv.es (P.C.); dtarrazo@fis.upv.es (D.T.-S.); sercasib@upv.es (S.C.-I.); crubiom@fis.upv.es (C.R.)

**Keywords:** fresnel zone plates, Bifocal Lenses, ultrasound focusing

## Abstract

In this work, we present a bifocal Fresnel zone plate (BiFZP) capable of generating focusing profiles with two different foci. The performance of the BiFZP is demonstrated in the ultrasound domain, with a very good agreement between the experimental measurements and the finite element method (FEM) simulations. This lens becomes an appealing alternative to other dual-focusing lenses, in which the foci location can only be set at a limited range of positions, such as M-bonacci zone plates. Moreover, the variation of the operating frequency has also been analyzed, providing an additional dynamic control parameter in this type of lenses.

## 1. Introduction

Acoustic focusing is a hot topic among the scientific community due to the multiple applications in both the industrial and healthcare sectors. In recent years, several techniques to focus acoustic waves have been reported, such as time-reversal mirrors, metasurfaces, or holographic lenses. Time-reversal mirrors (TRMs) take advantage of the time-reversal invariance of the wave equation to produce a focal spot at the target over an inhomogeneous acoustic medium [1,2,3,4]. On the other hand, acoustic metasurfaces employ 3D unit cells to generate a phase distribution at the output of the lens that ensures constructive interference at the desired focal length [5,6,7,8]. Another interesting approach to focusing ultrasonic beams is holographic lenses. Holographic lenses employ back-propagation techniques and iterative algorithms to produce an arbitrarily-shaped focused beam over a specific focal area [9,10,11,12].

However, all of these techniques have some drawbacks that may limit their application or their design. For instance, TRMs often require expensive phase-arrays to produce a narrow focal spot, acoustic metasurfaces need a complete 3D design of each one of their unit cells, and holographic lenses require a high computation burden to design the 3D height profile of the lens via an iterative back-propagation process. In this sense, Fresnel zone plates (FZPs) are a more conventional, yet effective, approach to achieve ultrasound focusing with high resolution.

FZPs are monofocal planar lenses formed by concentric rings of decreasing width, known as Fresnel regions. These devices have been widely used in various fields of physics, such as X-rays [13,14], optics [15,16,17], microwaves [18,19], and acoustics [20,21,22]. In [23], J. Kim et al. presented a method to achieve multifocal focusing profiles based on the phase conjugation principle and the phase-opposition blocking criterion used in conventional FZPs. Recently, novel lenses based on traditional FZPs have been introduced in optics [24,25,26,27] and later demonstrated in acoustics [28,29,30], which increases the versatility of these devices.

Among these novel designs, M-bonacci Zone Plates (MbZPs) stand out for their interesting bifocal focusing profiles [26,27], as they can produce two foci with equal acoustic intensities. However, MbZPs are only capable of generating foci at specific focal distances related with the M-bonacci sequence ratio, which significantly limits their flexibility [27]. The ratio between both foci in a MbZP remains constant when the order of the M-bonacci sequence is selected. As an example, in a Fibonacci ZP (2-order MbZP), the foci ratio is 1.618 and cannot be modified. Similarly, foci ratios of 1.192 and 1.078 are obtained when using MbZPs of orders 3 and 4, respectively. This work demonstrates a composite bifocal FZP, hereafter referred to as BiFZP, capable of producing a focusing profile with two foci. This BiFZP can be used in scenarios where MbZPs cannot achieve the required focal distances, and therefore it represents an interesting and flexible alternative to these kind of lenses.

## 2. BiFZP Lens Design

For plane wave incidence, the radii of the different Fresnel regions that form an FZP can be calculated as

(1) $$r_{n}=\sqrt{n\lambda F+{\left(\frac{n\lambda }{2}\right)}^{2}},$$

where *F* is the focal length, λ is the working wavelength and n=1,2,…,N, with *N* being the total number of Fresnel regions.

Once the FZP radii are calculated, the focusing profile of the lens is obtained by numerically computing the Rayleigh–Sommerfeld diffraction integral,

(2) $$I\left(z\right)=\frac{4\pi^{2}}{\lambda^{2}}{\left|\int_{0}^{r_{N}}p_{i}\left(r^{\prime }\right)t\left(r^{\prime }\right)\frac{e^{-jkR}}{R}\cos\left(n,R\right)r^{\prime }dr^{\prime }\right|}^{2},$$

where $r_{N}$ is the maximum radius of the lens, $r^{\prime }$ is the radial coordinate of the lens, k=2π/λ is the wavenumber, $p_{i}\left(r^{\prime }\right)$ is the incident pressure distribution, $t(r^{\prime })$ is the ZP transmittance function, $R=\sqrt{{\left(r^{\prime }\right)}^{2}+z^{2}}$, *z* is the axial coordinate and cos(n,R)=z/R, with *n* being the normal direction to the lens surface. For a Soret ZP, the transmittance function, also known as the pupil function, is 0 at the pressure blocking regions and 1 at the transparent regions.

**Figure 1:**
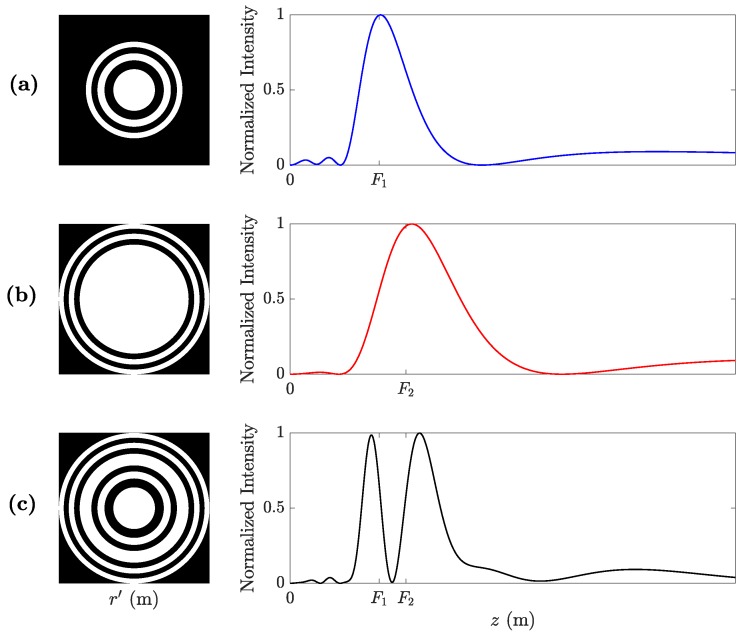
Fresnel zone plate (FZP) layouts and their corresponding normalized focusing profiles: (**a**) inner FZP, (**b**) outer FZP, and (**c**) bifocal Fresnel zone plate (BiFZP).

The BiFZP lens is obtained by combining the Fresnel regions of two conventional FZPs. The inner regions are designed to focus at a focal length $F_{1}$, while the outer regions are designed to focus at a different focal length of $F_{2}$. In this process, $F_{1}$ is selected to be closer to the lens, that is, $F_{1}<F_{2}$, which provides a better focusing performance. Figure 1 shows a schematic example of the lens design steps. Figure 1a depicts the layout of the inner Fresnel regions that have been designed using Equation (1) to focus at $F_{1}$ (left), and its corresponding focusing profile (right); while Figure 1b depicts the layout of the outer regions that focuses at $F_{2}$ (left) and its corresponding focusing profile (right). Here, each focusing profile is normalized to its maximum value. As can be observed from the figure, the focal length in this focusing profile, which theoretically corresponds to $F_{2}$, presents a slight shift due to the pupil effect of the layout depicted in Figure 1b (right), which has already been analyzed in [31].

Figure 1c (left) depicts the BiFZP layout, obtained by combining the inner and outer regions from the layouts of Figure 1a,b. As can be observed from Figure 1c (right), the focusing profile of the BiFZP achieves two isolated foci, which are encountered at the vicinity of their expected locations ($F_{1}$ and $F_{2}$). Both BiFZP foci present higher longitudinal resolution than those from their inner and outer FZP counterparts. Moreover, the focal lengths of the BiFZP foci are slightly shifted from their theoretical values $F_{1}$ and $F_{2}$. This fact is a consequence of the interference between the phase profiles of the inner and outer Fresnel regions that form the lens.

**Figure 2:**
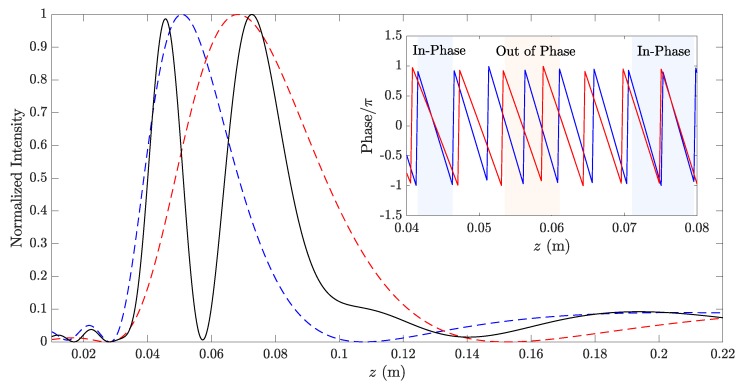
The bi-Fresnel zone plate focusing profile (black) compared to the inner (blue) and outer (red) focusing profiles. The phase of the inner (blue) and outer (red) profiles are shown in the inset.

In order to showcase this phenomenon, Figure 2 depicts the normalized focusing profile of a BiFZP alongside an inset of the phase profiles of the inner (blue color) and outer (red color) regions. The lens has been designed for focal distances $F_{1}=50$ mm, $F_{2}=65$ mm, frequency f=337.5 kHz, and a total number of Fresnel regions of N=9. The focusing profile has been computed using the Rayleigh–Sommerfeld diffraction integral. As can be observed from the figure, both foci are shifted towards the areas where the inner and outer phase profiles are in-phase ($F_{1}^{\prime }=45.6$ mm and $F_{2}^{\prime }=72.6$ mm), and the zero intensity value at z≅57 mm is achieved because both profiles are in phase opposition. Therefore, the BiFZP has to be designed considering the phase profiles of the inner and outer regions of the lens, as they play a very important role on the final focusing performance of the device.

**Figure 3:**
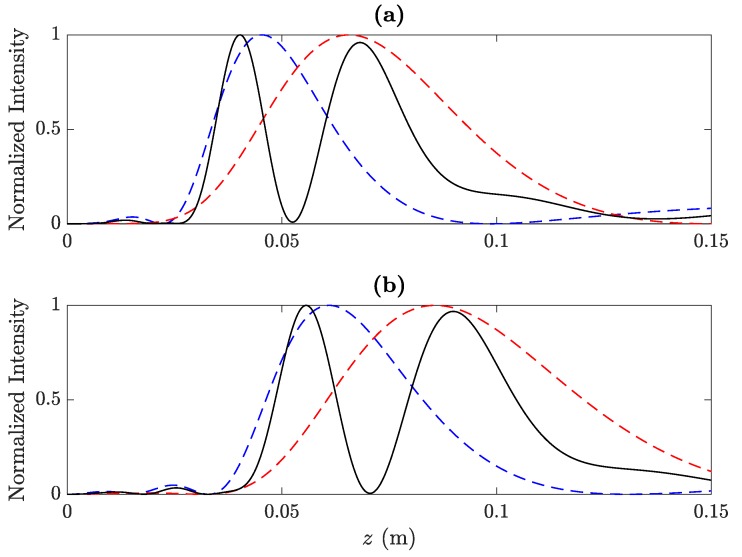
(**a**) The BiFZP focusing profile designed for $F_{1}^{\prime }=40$ mm and $F_{2}^{\prime }=68$ mm, and (**b**) the BiFZP focusing profile designed for $F_{1}^{\prime }=56$ mm and $F_{2}^{\prime }=90$ mm. In both cases, $f_{0}=250$ kHz.

In order to showcase the flexibility of the proposed design method, Figure 3 depicts two additional focusing profiles obtained using two different BiFZPs at 250 kHz. The BiFZP focusing profile depicted in Figure 3a (black line) is designed to focus at $F_{1}^{\prime }=40$ mm and $F_{2}^{\prime }=68$ mm. The monofocal FZPs that have been combined to build this BiFZP focus at $F_{1}=44.4$ mm (blue line) and $F_{2}=59.5$ mm (red line), respectively. Using a different BiFZP, Figure 3b shows a focusing profile (black line) with two foci located at $F_{1}^{\prime }=56$ mm and $F_{2}^{\prime }=90$ mm. The two corresponding monofocal FZPs, in this case, focus at $F_{1}=60.2$ mm (blue line) and $F_{2}=80.1$ mm (red line), respectively. As can be observed from Figure 3, both lenses achieve their expected design focal distances, showing, as a consequence of the phase profile interference, two well-defined foci with similar intensities and a sharp minimum very close to zero, which clearly isolates both foci.

In order to validate the design method, experimental measurements with a brass BiFZP have been carried out. In the experimental set-up, the distance between the transducer and lens is not large enough to consider the plane wave incidence. Therefore, the Fresnel radii have been calculated considering spherical wave incidence [32], that is,

(3) $$d+F+\frac{n\lambda }{2}=\sqrt{d^{2}+r_{n}^{2}}+\sqrt{F^{2}+r_{n}^{2}},$$

where *d* is the distance between the transducer and lens.

The final design of the lens is obtained using a two-step process. For the first step, ideal simulations are carried out using MATLAB to numerically calculate the Rayleigh–Sommerfeld integral (Equation (2)), in order to obtain the focal lengths, so that the resulting focusing profile exhibits two well-defined foci with an intermediate zero value. Once $F_{1}$ and $F_{2}$ are obtained for the design frequency, iterative finite element method (FEM) simulations are carried out using COMSOL Multiphysics, in order to optimize the number of inner and outer Fresnel regions that provide a focusing profile with balanced intensities at both foci. FEM simulations solve the wave equation in each medium (water and brass), taking into account acoustic solid–liquid interactions, and therefore providing a better characterization of the lens performance. It is worth noting that the number of inner and outer Fresnel regions can only be increased or decreased in discrete steps, which means that the focal intensities will also increase or decrease in discrete levels.

**Figure 4:**
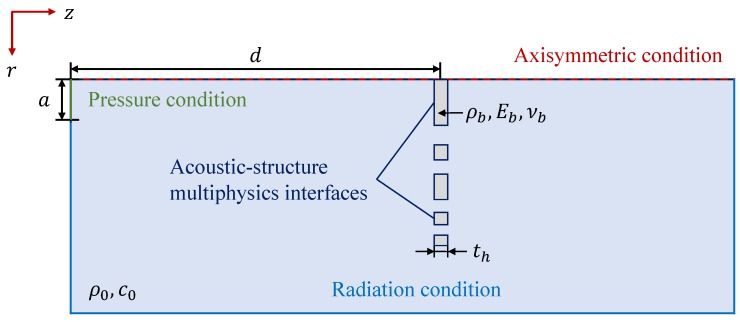
Scheme of the finite element method (FEM) model.

Figure 4 shows a scheme of the axisymmetric FEM model employed in the simulation. The lens is modeled as a linear elastic material immersed in water, with $t_{h}$ as the thickness of the lens. An acoustic-structure multiphysics interface is established at the lens boundaries in order to account for the solid–liquid wave interactions. The transducer is modeled as a pressure condition of length, *a*, with *a* as the active radius of the transducer, separated a distance, *d*, from the lens, and a radiation condition is set at the outer boundaries in order to avoid reflections. Table 1 shows the acoustic properties of the materials used in the simulations. In this table, ρ represents the material density, *E* the Young modulus, ν the Poisson’s ratio, and *c* the speed of sound.

## 3. Experimental Results and Discussion

The experimental set-up consists of a 3D underwater automated positioning system with a spatial resolution of 1×1×1 mm${}^{3}$. An Imasonic piston transducer with a 30 mm active diameter and a central frequency of 250 kHz is used as the emitter. The feeding signal of the transducer is generated using a Panametrics 5077PR pulser.

**Figure 5:**
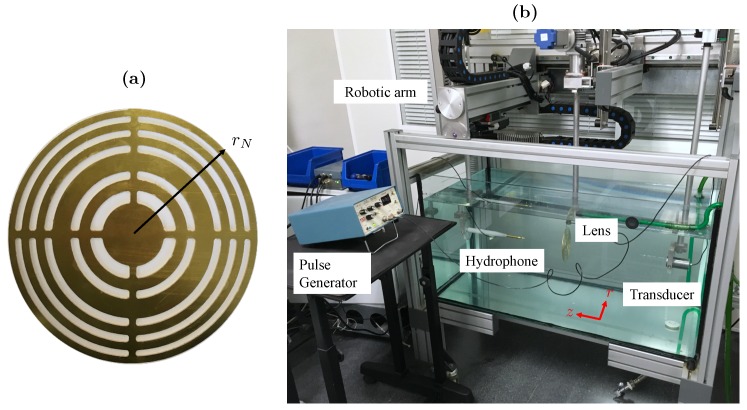
(**a**) Manufactured BiFZP lens made of brass and (**b**) the experimental set-up with the lens.

The receiver consists of a needle hydrophone from Precision Acoustics Ltd. with a 1.5 mm active diameter and a −4 dB bandwidth ranging from 200 kHz to 25 MHz. The needle hydrophone is connected to a low noise preamplifier and then to a digital oscilloscope from Pico Technology. The manufactured BiFZP is made of brass with a thickness of $t_{h}=1$ mm and is located at a distance d=350 mm from the piston transducer. The lens has been designed for focal lengths $F_{1}=50$ mm and $F_{2}=70$ mm, and a design frequency of 250 kHz. The BiFZP outermost radius is $r_{N}=66.71$ mm. Figure 5a,b show a picture of the manufactured brass BiFZP and the experimental set-up, respectively.

**Figure 6:**
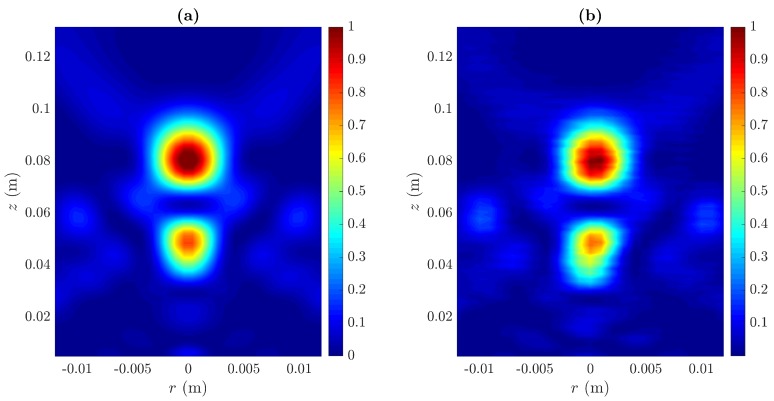
(**a**) Simulated and (**b**) measured normalized acoustic intensity maps for the manufactured BiFZP lens.

Figure 6a,b depict the simulated and measured acoustic intensity maps, respectively. Each map is normalized to its maximum value. As can be observed, the FEM and experimental results are in good agreement, and the manufactured BiFZP provides a bifocal focusing profile with two well-defined foci.

**Figure 7:**
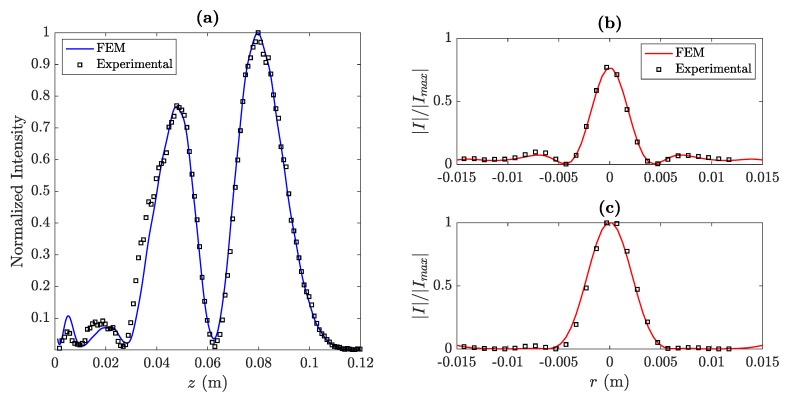
(**a**) Measured (black squares) and simulated (blue line) focusing profiles (longitudinal cuts) for the manufactured BiFZP lens. (**b,c**) Measured (black squares) and simulated (red lines) transversal cuts at $z=F_{1}^{\prime }$ (**b**) and $z=F_{2}^{\prime }$ (**c**).

Figure 7 shows the longitudinal and transversal cuts of the acoustic intensity maps shown in Figure 6. Experimental measurements are represented using black squares, whereas the simulated results are depicted as solid lines in both longitudinal (blue) and transversal (red) cuts.

The longitudinal cut shown in Figure 7a corresponds to the BiFZP focusing profile at r=0. As expected, the focal lengths are shifted from the theoretical values ($F_{1}=50$ mm and $F_{2}=70$ mm) to $F_{1}^{\prime }=47.8$ mm and $F_{2}^{\prime }=79.8$ mm, as a consequence of the interference between the inner and outer phase profiles. As can be observed from the figure, both foci are clearly isolated and a very well-defined null can be found at z=62.8 mm.

The transversal cuts corresponding to $F_{1}^{\prime }$ and $F_{2}^{\prime }$ are shown in Figure 7b,c, respectively. As can be observed, there is a slight decrease in the lateral resolution at the second focus ($F_{2}$), due to the fact that it is located further apart from the BiFZP. This effect becomes more noticeable when the distance between the two foci of the BiFZP lens is further increased. As can be observed from Figure 7, the experimental measurements are in good agreement with the simulation results.

**Figure 8:**
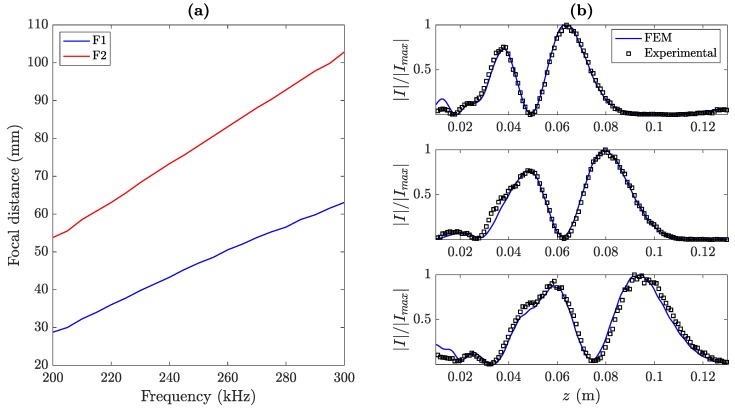
(**a**) BiFZP focal distance for $F_{1}^{\prime }$ (blue) and $F_{2}^{\prime }$ (red) as a function of the operating frequency and (**b**) BiFZP focusing profiles at 220 kHZ (top), 250 kHz (middle) and 280 kHz (bottom).

Figure 8a shows the focal length dependence on the operating frequency. As can be observed from the figure, both focal lengths shift linearly with frequency, which agrees with the results reported in previous works [29,33]. Moreover, Figure 8b depicts the simulated and measured focusing profiles for three different frequencies: 220 kHz (top), 250 kHz (middle), and 280 kHz (bottom). Thus, when the operating frequency is increased, both foci are shifted away from the lens, and therefore the operating frequency becomes an interesting parameter to dynamically control the focusing profile of the lens.

## 4. Conclusions

In this work, a design method to obtain bifocal focusing profiles by combining two conventional monofocal FZPs is presented. The experimental results are discussed, showing good agreement with numerical simulations and demonstrating that BiFZPs can become an appealing alternative to MbZPs in scenarios where MbZPs cannot achieve the required focal lengths. In addition, BiFZPs present a linear dependence on the operating frequency, which can become an interesting parameter to dynamically tune the focal lengths once the lens is manufactured.

## Figures and Tables

**Table 1:** Acoustic properties used in FEM simulations.

Material	Property
	$\rho_{b}=8500$ kg/m${}^{3}$
Brass	$E_{b}=104$ GPa
	$\nu_{b}=0.37$
Water	$\rho_{0}=1000$ kg/m${}^{3}$
	$c_{0}=1500$ m/s
